# Effectiveness and Safety of Off-label Dosing of Non–vitamin K Antagonist Anticoagulant for Atrial Fibrillation in Asian Patients

**DOI:** 10.1038/s41598-020-58665-5

**Published:** 2020-02-04

**Authors:** Kwang-No Lee, Jong-Il Choi, Ki Yung Boo, Do Young Kim, Yun Gi Kim, Suk-Kyu Oh, Yong-Soo Baek, Dae In Lee, Seung-Young Roh, Jaemin Shim, Jin Seok Kim, Young-Hoon Kim

**Affiliations:** 1 Division of Cardiology, Department of Internal Medicine, Korea University College of Medicine and Korea University Medical Center, Seoul, Republic of Korea; 2 Division of Cardiology, Department of Internal Medicine, Inha University Hospital, Incheon, Republic of Korea; 3 Division of Cardiology, Department of Internal Medicine, Chungbuk National University Hospital, Cheongju, Republic of Korea

**Keywords:** Atrial fibrillation, Stroke

## Abstract

Non–vitamin K antagonist anticoagulants (NOACs) have been used to prevent thromboembolism in patients with atrial fibrillation (AF) and shown favorable clinical outcomes compared with warfarin. However, off-label use of NOACs is frequent in practice, and its clinical results are inconsistent. Furthermore, the quality of anticoagulation available with warfarin is often suboptimal and even inaccurate in real-world data. We have therefore compared the effectiveness and safety of off-label use of NOACs with those of warfarin whose anticoagulant intensity was accurately estimated. We retrospectively analyzed data from 2,659 and 3,733 AF patients at a tertiary referral center who were prescribed warfarin and NOACs, respectively, between 2013 and 2018. NOACs were used at off-label doses in 27% of the NOAC patients. After adjusting for significant covariates, underdosed NOAC (off-label use of the reduced dose) was associated with a 2.5-times increased risk of thromboembolism compared with warfarin, and overdosed NOAC (off-label use of the standard dose) showed no significant difference in either thromboembolism or major bleeding compared with warfarin. Well-controlled warfarin (TTR ≥ 60%) reduced both thromboembolism and bleeding events. In conclusion, the effectiveness of NOACs was decreased by off-label use of the reduced dose.

## Introduction

Phase III randomized controlled trials for non–vitamin K antagonist anticoagulants (NOACs) in patients with non-valvular atrial fibrillation (NVAF) have demonstrated, at a minimum, their noninferiority to warfarin in preventing thromboembolism and bleeding events^1–4^. Recent real-world studies have also shown that both the standard and reduced doses of NOACs are effective and safe compared with warfarin^5–7^. However, those real-world data showed a high prescription rate for the reduced dose, 50–90% of all NOAC prescriptions, which might be underdosing a considerable number of patients^5–7^. The off-label dosing of NOACs in practice, mostly at the reduced dose, has been reported in up to 50% of AF patients receiving a NOAC^8–10^.

Clinical results from off-label dosing of NOACs have been inconsistent. In the ORBIT-AF II cohort, off-label dosing was associated with an increased risk of adverse events compared with on-label dosing^8^. On the contrary, the off-label use of a reduced dose in Asian patients was found to be safe and effective compared with warfarin^11,12^. However, suboptimal anticoagulation is frequently reported with warfarin in Asian patients, which could affect the comparison between NOACs and warfarin^13–15^. Therefore, our objective in this study was to compare the effectiveness and safety of NOACs (by dose and adherence with the drug label) with that of warfarin after adjusting for heterogeneous covariates.

## Methods

### Data collection

We collected data from the electronic medical records of all patients who had been prescribed warfarin, dabigatran, rivaroxaban, or apixaban from January 2013 to May 2017 or edoxaban from February 2016 to June 2018 for AF at least once. The Korean Ministry of Food and Drug Safety (MFDS) approved dabigatran for the prevention of stroke or systemic embolism in patients with NVAF in 2011. Rivaroxaban and apixaban have been available in clinical practice since January 2013, and edoxaban has been available since February 2016.

We collected data on demographics, comorbidities, concomitant drug use, blood laboratory findings, echocardiographic findings, surface 12-lead electrocardiography (ECG), hospitalization, thromboembolic or bleeding events, and mortality. The serum creatinine clearance (sCCr) was determined by the Cockcroft-Gault formula using the most recent serum creatinine value before inclusion in this study.

The duration of anticoagulant therapy was calculated based on the prescription date and total number of days of treatment. Data were excluded if treatment was discontinued for more than 30 days. Time-varying drug adherence for a received anticoagulant drug was estimated using the proportion of days covered (PDC), which was defined as the ratio of the number of days a patient was actually on treatment to the number of days the patient should have been given the drug during the study follow-up period^16^. Patients were considered adherent if they met the PDC threshold (≥80%) based on the median number of 5 (2, 10) prescriptions and median interval length of 12 (1, 63) days between one prescription and the next. The time in therapeutic range (TTR) for warfarin was calculated using the Rosendaal method with an international normalized ratio between 2.0 and 3.0. It was calculated by means of linear interpolation to assess the adequacy of anticoagulation^17^. Concomitant drug use was defined as concurrent use of medication for ≥80% of the follow-up period. The Ethics Committee of the Korea University Anam Hospital Institutional Review Board approved this study and waived informed consent. All patient records and medical information were anonymized prior to analysis. The protocol of the current study was consistent with the ethical guidelines of the 2008 Helsinki Declaration.

### Study design

**Figure 1:**
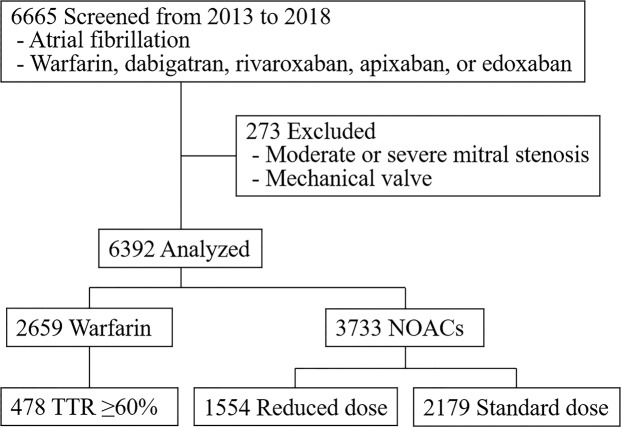
Flow chart showing the selection of study subjects.

This was a retrospective observational study conducted at a single center. Patients diagnosed with AF by ECG analysis who were prescribed an anticoagulant (warfarin, dabigatran, rivaroxaban, apixaban, or edoxaban) were included. We excluded patients who had moderate or severe mitral stenosis or a mechanical prosthetic heart valve (Fig. 1).

Off-label use of a standard dose was defined as a standard dose of NOAC prescribed when the indication was for a reduced dose, and off-label use of a reduced dose was defined as a reduced dose prescribed when the indication was for a standard dose, according to the dose and summary of product characteristics approved by the Korean MFDS (Supplementary Table S1). Therefore, NOAC use was classified as on-label use of a reduced (ON-R) or standard dose (ON-S) and off-label use of a reduced (OFF-R) or standard dose (OFF-S). The standard dosage and administration regimes were dabigatran (150 mg) twice daily, rivaroxaban (20 mg) once daily, apixaban (5 mg) twice daily, and edoxaban (60 mg) once daily. The reduced dosage and administration regimes were dabigatran (110 mg) twice daily, rivaroxaban (15 mg) once daily, apixaban (2.5 mg) twice daily, and edoxaban (30 mg) twice daily. Because dabigatran has no pre-specified dose reduction criteria, we defined the condition for a reduced dose of dabigatran as an sCCr level ≥30 and <50 mL/min.

The outcomes of interest for effectiveness were thromboembolic events during follow-up: confirmed ischemic stroke, systemic thromboembolism, myocardial infarction, or intracardiac thrombi. All ischemic stroke events were confirmed by diagnostic imaging that excluded transient ischemic attacks. The measure of safety outcome was defined as an overt bleeding event consistent with the International Society on Thrombosis and Hemostasis definition of major bleeding in non-surgical patients^18^: (1) fatal bleeding, (2) symptomatic bleeding in a critical area or organ (intracranial, intraspinal, intraocular, retroperitoneal, intra-articular or pericardial, or intramuscular with compartment syndrome), (3) a fall in hemoglobin level of 20 g/L or more, or (4) transfusion ≥ 2 units of whole blood or red cells. Outcomes were adjudicated based on the medical records including imaging and patient notes.

### Statistical analysis

The baseline characteristics of patients, divided by anticoagulant treatment, were tested for differences between groups using the Student’s t-test for continuous variables with equal variances, Welch’s t-test for continuous variables with unequal variances, Mann-Whitney U-test for data with a non-normal distribution, and the chi-square test for categorical variables. Drug exposure was defined as a time-dependent variable^19^. To minimize bias from time-varying exposure, patients who switched to different agents required different exposure periods. The cumulative risk of an event during the anticoagulation period was estimated by a Kaplan-Meier analysis and compared using the log-rank test. The relative hazard ratios (HRs) for thromboembolism and major bleeding events were determined with Cox proportional hazards regression models using the following covariates: age, sex, comorbidities (hypertension (HTN), diabetes mellitus (DM), and congestive heart failure (CHF)), prior thromboembolism (ischemic stroke, myocardial infarction, systemic embolism, deep vein thrombosis, or intracardiac thrombus), concomitant antiplatelet drugs, sCCr, and the anteroposterior diameter of the left atrium on echocardiography. The proportional hazard assumption was evaluated using log minus log survival curves that were approximately parallel over the follow-up period. We used Firth’s penalized maximum likelihood bias reduction method for the Cox regression to obtain HRs in the survival analyses^20^. Comparisons between the OFF-R and ON-S and OFF-S and ON-R groups used the Bonferroni correction for multiple testing. Odds ratios for associations with prescribing OFF-R compared with ON-S were determined with a logistic regression model after adjusting for differences in sex, age, weight, height, HTN, DM, CHF, prior thromboembolism, concomitant antiplatelet drug use, and serum creatinine. To determine the influence of the dosing criteria for NOAC, a sensitivity analysis was performed with simplified criteria (sCCr only). In that analysis, the ranges for a reduced dose of NOACs were 30–50 mL/min for dabigatran, 15–30 mL/min for apixaban, and 15–50 mL/min for rivaroxaban and edoxaban. All statistical analyses were performed using SPSS version 24 (IBM Corp. in Armonk, NY, USA) and SAS version 9.4 (SAS Institute, Inc., Cary, NC, USA). All tests of significance were two-tailed. A *p*-value < 0.05 indicated statistical significance.

## Results

### Baseline characteristics

We retrospectively evaluated data from 6,665 NVAF patients who were prescribed anticoagulants. We excluded 273 patients who had mitral stenosis or a mechanical heart valve. In the end, we included 2,659 patients who were using warfarin and 3,733 patients who were using NOACs (Fig. 1). The mean age of the included patients was 66.8 ± 11.7 years, and 34.8% of the participants were female. The median CHA_2_DS_2_-VASc score of the included patients was 3 (2 to 4). The median TTR in the warfarin group was 30.4% (6.0 to 53.4), calculated using a median of 8 (4 to 14) blood samples. A total of 478 (18.0%) patients who had a TTR ≥60% were defined as the well-controlled warfarin group. The median TTR in the well-controlled warfarin group was 71.8% (65.8 to 83.5). The percentage of patients considered adherent to their prescribed drug, with a PDC ≥80%, was more than 85% in all groups (Table 1). The all NOAC group was older with a higher rate of prior ischemic events and higher proportion of women, which coincided with a higher CHA_2_DS_2_-VASc score, than the all warfarin group. A concomitant antiplatelet drug was used by 11.1% and 6.3% of patients taking warfarin and a NOAC, respectively.

**Table 1:** Baseline characteristics of patients who received warfarin or a NOAC for AF.

	All Warfarin (n = 2,659)	All NOAC (n = 3,733)	Reduced Dose of a NOAC (n = 1,554)^†^		Standard Dose of a NOAC (n = 2,179)^‡^	
			On-label (n = 777)	Off-label (n = 733)	On-label (n = 1,873)	Off-label (n = 226)
Age, years	65.3 ± 11.9	68.0 ± 11.4*	77.3 ± 8.1*	70.9 ± 8.2*,**	62.4 ± 10.7*	73.0 ± 9.2*
Female	820 (30.8)	1,404 (37.6)*	474 (61.0)*	273 (37.2)*,**	496 (26.5)*	114 (50.4)*
Height, cm	164.3 ± 9.4	163.4 ± 9.5*	156.5 ± 8.8*	163.1 ± 8.1*,**	166.9 ± 8.6*	159.4 ± 8.8*
Weight, kg	67.7 ± 12.1	66.6 ± 12.0*	56.8 ± 9.7*	67.7 ± 10.2**	71.0 ± 11.2*	59.3 ± 8.5*
HTN	1,980 (74.5)	2,837 (76.0)	650 (83.7)*	626 (85.4)*,**	1,315 (70.2)*	176 (77.9)
DM	717 (27.0)	1,015 (27.2)	239 (30.8)	241 (32.9)*,**	452 (24.1)*	70 (31.0)
CHF	865 (32.5)	862 (23.1)*	303 (39.0)*	202 (27.6)*,**	282 (15.1)*	68 (30.1)
Any prior TE^§^	600 (22.6)	1,029 (27.6)*	311 (40.0)*	218 (29.7)*,**	414 (22.1)	82 (36.3)*
CHA_2_DS_2_-VASc score^¶^	3 (1, 4)	3 (2, 4)*	4 (3, 6)*	3 (2, 5)*,**	2 (1, 3)*	4 (3, 5)*
Concomitant AP	296 (11.1)	234 (6.3)*	70 (9.0)	84 (11.5)**	65 (3.5)*	15 (6.6)*
sCCr, mL/min	68.6 ± 27.2	66.6 ± 24.7*	43.9 ± 14.7*	64.5 ± 18.4*,**	78.6 ± 23.0*	51.2 ± 16.6*
LV EF, %	51.6 ± 10.0	52.7 ± 8.7*	51.9 ± 10.0	52.4 ± 8.9	53.2 ± 7.9*	51.9 ± 9.8
LAD	44.6 ± 7.1	44.0 ± 7.0*	44.7 ± 7.7	45.5 ± 7.3*,**	43.2 ± 6.4*	44.0 ± 7.3
PDC, %	92.0 ± 17.0	91.4 ± 19.1	91.9 ± 17.9	92.5 ± 17.4**	90.8 ± 19.9*	90.0 ± 23.3
≥80%	2,152 (87.6)	2,760 (86.6)	595 (88.1)	535 (88.6)**	1,384 (85.1)*	170 (86.3)

Data are presented as the mean ±standard deviation, median (25^th^, 75^th^ percentiles), or number (%).

*p < 0.05 with the all warfarin group.

**p < 0.05 between the off-label reduced dose and the on-label standard dose of NOAC groups.

^†^There were no label indication data for 44 patients.

^‡^There were no label indication data for 80 patients.

^§^Ischemic stroke, myocardial infarction, peripheral arterial disease, peripheral venous thromboembolism, and pulmonary embolism.

^¶^One point each for congestive heart failure, hypertension, age of 65–74 years, diabetes mellitus, and vascular disease (myocardial infarction or peripheral arterial disease), and two points for age of 75 years or older and a previous stroke.

Abbreviations: AP, antiplatelet drug; CHF, congestive heart failure; DM, diabetes mellitus; NOAC, non–vitamin K antagonist anticoagulant; HTN, hypertension; LAD, left atrial anteroposterior dimension; LV EF, left ventricular ejection fraction; MI, myocardial infarction; PDC, proportion of days covered; sCCr, serum creatinine clearance; TE, thromboembolism; TTR, time in therapeutic range.

**Figure 2:**
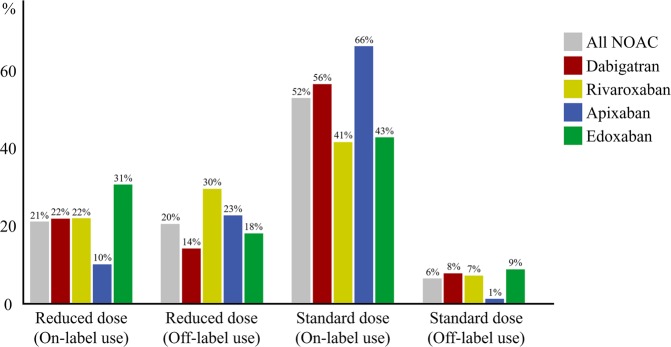
NOAC use by dose and adherence to label indications.

Among the patients on NOAC treatment, 41.8% received a reduced dose. Those patients had more covariates related to the risk of thromboembolism and bleeding than either the group treated with warfarin or the group that received a standard dose of a NOAC. Reduced and standard doses of NOAC were used as off-label indications in 20.3% and 6.3% of patients receiving a NOAC, respectively (Fig. 2).

### Thromboembolism

**Figure 3:**
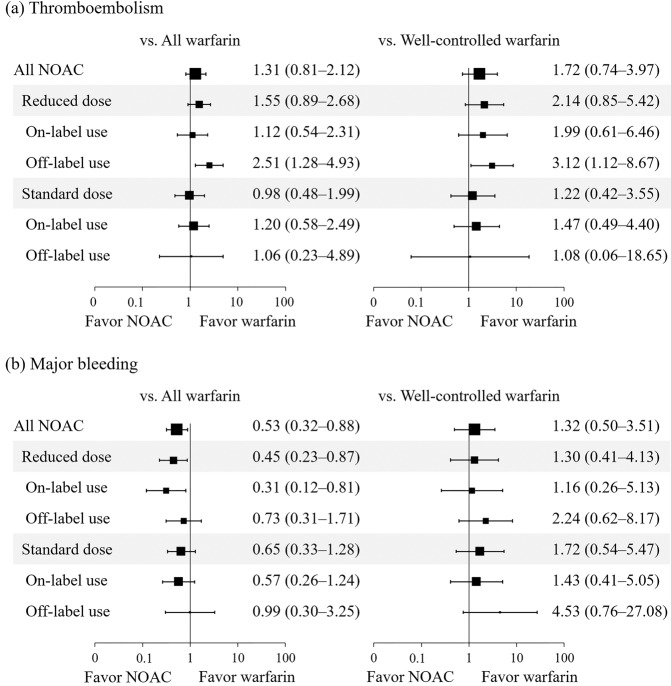
Forest plot of the adjusted hazard ratio and 95% confidence intervals for thromboembolism (**a**) and major bleeding (**b**) in the NOAC group compared with the warfarin groups with any TTR and TTR ≥60%. Adjusted factors were age, sex, hypertension, diabetes mellitus, congestive heart failure, prior thromboembolism, concomitant antiplatelet drugs, serum creatinine clearance, and left atrial anteroposterior diameter.

**Figure 4:**
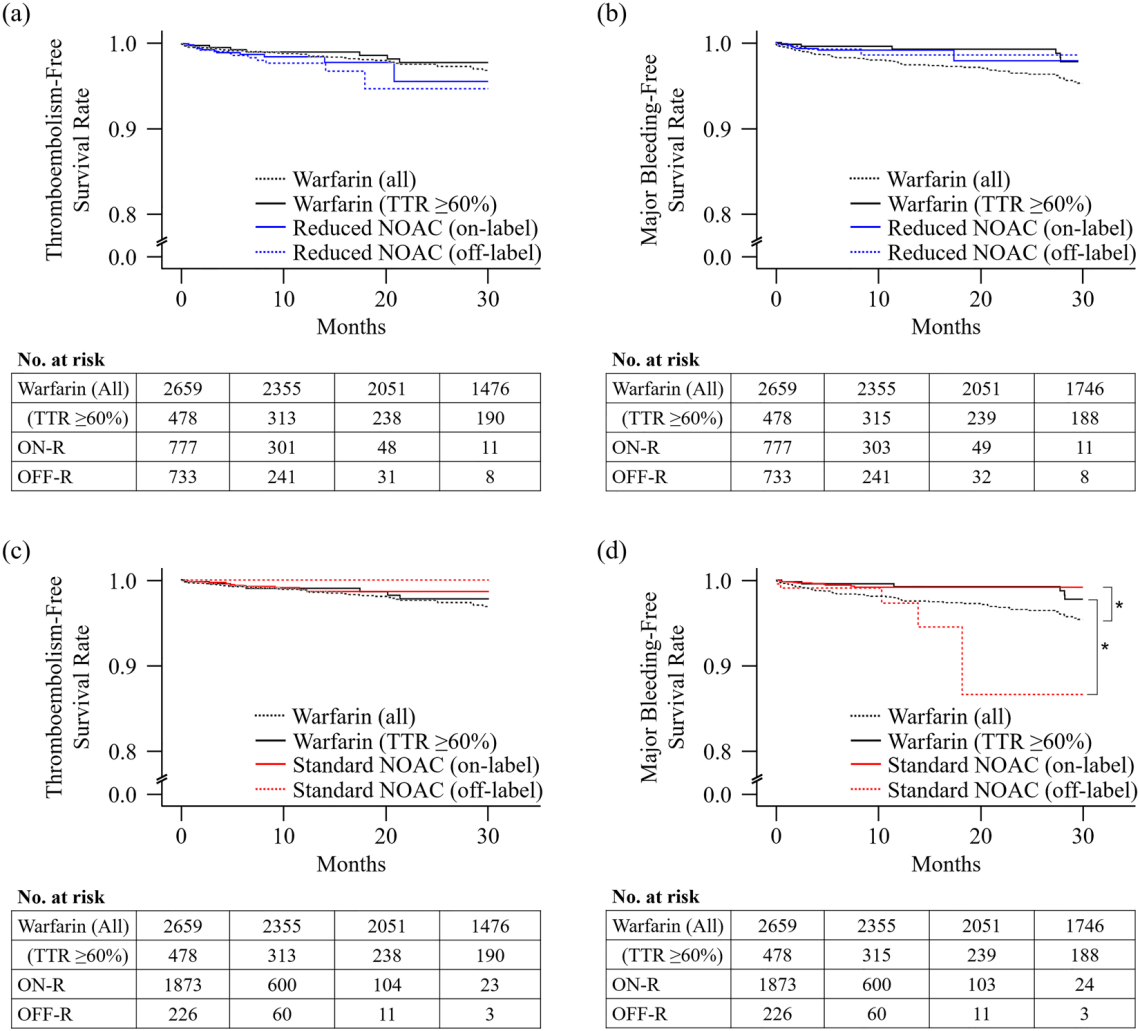
Kaplan-Meier curves of the time to first event by anticoagulation treatment: thromboembolism on reduced dose (**a**), major bleeding on reduced dose (**b**), thromboembolism on standard dose (**c**), and major bleeding on standard dose of a NOAC (**d**). Asterisks represent statistical significance compared with the well-controlled warfarin group by the log-rank test.

During a median follow-up of 6.3 and 9.9 months, 38 (1.02%) and 47 (1.77%) patients experienced thromboembolism at a rate of 1.13% and 1.14% of patients/year in the NOAC and warfarin groups, respectively. The underdosed NOAC (OFF-R) group had an increased risk of thromboembolism compared with the all warfarin group (adjusted HR [aHR] = 2.51; 95% confidence interval [CI] = 1.28–4.93) and the well-controlled warfarin group (aHR = 3.12; 95% CI = 1.12–8.67) (Fig. 3, Supplementary Table S2, and Supplementary Table S3). After correction for multiple testing by means of the Bonferroni correction, we observed a significant increase in thromboembolic events in the OFF-R NOAC group compared with the warfarin group (Supplementary Table S2). The OFF-S group had no significant difference in the risk of thromboembolism compared with any warfarin group. In the Kaplan-Meier analysis, none of the NOAC groups had a significant difference in event-free survival compared with the well-controlled warfarin group (Fig. 4a,c). However, the OFF-R group showed a trend toward decreased event-free survival (log-rank p = 0.06) (Fig. 4a).

### Major bleeding

During a median follow-up of 6.3 and 9.8 months, 30 (0.80%) and 74 (2.78%) patients experienced major bleeding at a rate of 0.44% and 0.95% of patients/year in the NOAC and warfarin groups, respectively. The ON-S group in the unadjusted model (unadjusted HR = 0.36; 95% CI = 0.19–0.69) and the ON-R group in the adjusted model (aHR = 0.31; 95% CI = 0.12–0.81) had a decreased risk of events compared with the all warfarin group but not compared with the well-controlled warfarin group (Fig. 3, Supplementary Table S2, Supplementary Table S3, and Fig. 4b). The OFF-S group had a higher risk of events than the well-controlled warfarin group in the unadjusted model (unadjusted HR = 6.16; 95% CI = 1.60–23.62) but not in the adjusted model (Fig. 3, Supplementary Table S2, and Supplementary Table S3). In the Kaplan-Meier analysis, the OFF-S group showed decreased event-free survival compared with the well-controlled warfarin group (log-rank p < 0.01) (Fig. 4d).

### Sensitivity analysis

A sensitivity analysis using only the sCCr criterion for dosing of the NOACs showed results similar to those obtained from the original analysis (Supplementary Table S4 and Supplementary Figure S1).

### Patients vulnerable to underdosed NOAC

**Figure 5:**
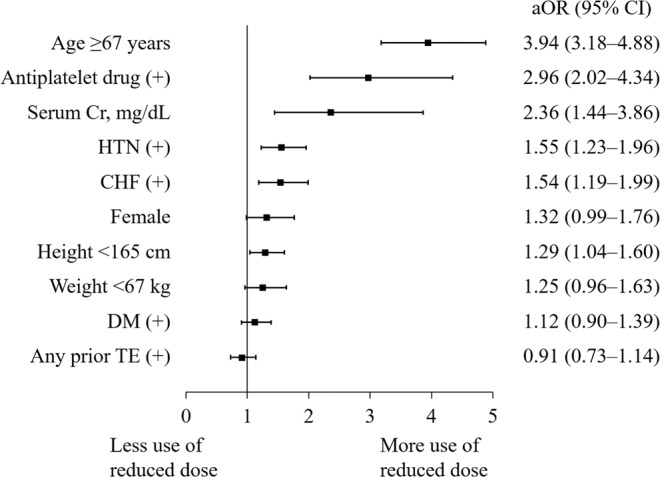
Forest plot of adjusted odds ratios (aORs) and 95% confidence intervals (CIs) for OFF-R dosing in patients appropriate for the standard dose of a NOAC. ORs are adjusted for the variables shown in the figure.

Age ≥ 67 years was the most potent risk factor for OFF-R dosing in patients appropriate for a standard dose of a NOAC, followed by concomitant use of an antiplatelet drug, serum creatinine, HTN, CHF, female sex, and height <165 cm (Fig. 5).

## Discussion

In this study, after adjusting for multiple baseline covariates, we compared the effectiveness and safety of NOACs with those of warfarin. Off-label dosing was used in a relatively high percentage of the patients who received a reduced NOAC dose. That use was associated with older age, concomitant antiplatelet drug use, and decreased renal function. Compared with warfarin, NOACs had similar effectiveness, except for OFF-R dosing, which was associated with a higher thromboembolic risk. The relative safety of NOACs depended on the adequacy of anticoagulation achieved with warfarin. The NOAC groups had a lower bleeding risk than the all warfarin group but not than the well-controlled warfarin group.

Recently, the effectiveness and safety of NOACs in the real world have been reported using nationwide population-based data^6,21–24^. The risks of thromboembolism and major bleeding in patients taking a NOAC were found not to differ or to decrease compared with those in patients taking warfarin^6,21–24^. In those studies, the annual incidence rates for ischemic stroke and intracranial hemorrhage were <4% and <2%, respectively. and the differences of those event rates in the NOAC and warfarin groups were less than 3%.

The smaller the difference between the events of interest in the treatment groups, the larger the required sample size will be. However, large datasets can have systematic biases^25^. In particular, missing information impedes the analysis of suspected associations. Therefore, we examined appropriate drug use and adherence, and we assessed the intensity of anticoagulation in patients on warfarin therapy by TTR using the Rosendaal method. In this study, the risk of thromboembolism in patients who were taking warfarin did not differ significantly from that in patients using a NOAC. However, the risk among warfarin patients decreased as the intensity of anticoagulation improved. This finding is similar to the results of previous studies^26,27^.

There are concerns about anticoagulation treatment using a vitamin–K antagonist in Asian countries. Phase III trials evaluating NOAC use have demonstrated that the annual incidence rates of ischemic stroke or systemic embolism and major bleeding with warfarin are higher in Asian patients than in non-Asian patients^15,28–30^. A possible explanation for that higher rate of thromboembolism is poor control of anticoagulation, as represented by a low TTR^13–15^. Our finding that the incidence rate of thromboembolism decreased as TTR increased in patients taking warfarin supports that explanation. Another concern is a bleeding tendency at the traditional target international normalized ratio (INR) range of 2.0−3.0, which was determined mainly in non-Asian patients. Among elderly Japanese people, a lower INR range of 1.6−2.6 has been reported to decrease the number of serious bleeding events^31,32^, which has been reflected in the Japanese AF guideline^33^.

A similar tendency of physicians to reduce the intensity of anticoagulant therapy for NOAC prescriptions has been observed in Asian countries. Whereas OFF-R doses were prescribed to 9–12% of patients receiving a NOAC in the United States^8,34^, close to 90% of the nationwide cohort participants in Taiwan were prescribed a reduced dose^24^. In the present study, old age was the most potent risk factor for an OFF-R prescription. Although the Cockcroft-Gault formula has a negative association with age, many elderly patients with normal renal function did not necessarily require a dose reduction.

The NOAC doses used in phase III trials were determined from the pharmacokinetic and pharmacodynamic properties that accompanied proportional increases in dose^35–37^. Plasma concentrations of NOACs depend on renal function and correlate with the inhibition of coagulation factors. Although the degree of inhibition expected with the use of drug-specific standard doses showed non-inferiority to warfarin in phase III trials, the clinical effectiveness of the probable lower inhibition provided by the reduced dose was not determined. The OFF-R group showed no significant difference in the risk of thromboembolism or bleeding compared with the ON-S group in the United States^8,34^. In Japan, OFF-R dosing of rivaroxaban showed non-inferiority to the well-controlled warfarin group for both thromboembolism and bleeding^11^. A Taiwanese nationwide cohort study showed that dabigatran and rivaroxaban accounted for 90% of the agents used at a reduced dose and significantly decreased the risk of thromboembolism and intracranial hemorrhage compared with warfarin^24^. In the present study, however, the OFF-R group had an increased a risk of thromboembolism compared with warfarin, whereas the ON-S group had no significant differences in thromboembolism or major bleeding compared with warfarin.

This study has some limitations. First, it was conducted at a single tertiary referral center. Patient who experienced any thromboembolic or bleeding event might have been selectively referred from their primary clinic, which means that our study population might not be representative of the population we intended to analyze. Second, the data were collected retrospectively. Even though consecutive patients were meticulously reviewed with caution to prevent loss of information, a substantial loss to follow-up could have decreased the accuracy of our estimated event-free survival with the possibility of missing of endpoints if patients were referred to another hospital. However, our inclusion of additional data, including echocardiographic parameters and laboratory findings, could help to expand the results of previous large-scale, real-world studies. Third, the total number of patients might be not sufficient to robustly compare the various subgroups of NOACs because events occurred rarely. Fourth, we included not only patients who were prescribed a NOAC or warfarin as the first anticoagulant, but also those who were switched to other anticoagulants. This might have increased the event rates compared with the phase III trials that included only anticoagulation-naive patients. To minimize selection bias, we applied our inclusion criteria equally to both groups. Fifth, our short follow-up duration decreased the number of events and changed the strength of risk for the study endpoint. Sixth, the NOAC groups included four drugs, so those results might depend on their respective proportions, and the results from the warfarin group, which had a low number of adequately treated patients, might depend on their intensity of anticoagulation.

## Conclusions

This study has demonstrated that the on-label use of NOAC in Asians with AF was effective and safe compared with warfarin in real-world practice. In the reduced dose group, the off-label dosing increased the risk of thromboembolism.

## Supplementary information

Supplementary Tables and Figure.

## References

[1] Connolly SJ (2009). Dabigatran versus warfarin in patients with atrial fibrillation. N. Engl. J. Med..

[2] Granger CB (2011). Apixaban versus warfarin in patients with atrial fibrillation. N. Engl. J. Med..

[3] Patel MR (2011). Rivaroxaban versus warfarin in nonvalvular atrial fibrillation. N. Engl. J. Med..

[4] Giugliano RP (2013). Edoxaban versus warfarin in patients with atrial fibrillation. N. Engl. J. Med..

[5] Cha MJ (2017). Effectiveness and Safety of Non-Vitamin K Antagonist Oral Anticoagulants in Asian Patients With Atrial Fibrillation. Stroke.

[6] Larsen TB, Skjoth F, Nielsen PB, Kjaeldgaard JN, Lip GY (2016). Comparative effectiveness and safety of non-vitamin K antagonist oral anticoagulants and warfarin in patients with atrial fibrillation: propensity weighted nationwide cohort study. BMJ.

[7] Maura G (2015). Comparison of the short-term risk of bleeding and arterial thromboembolic events in nonvalvular atrial fibrillation patients newly treated with dabigatran or rivaroxaban versus vitamin K antagonists: a French nationwide propensity-matched cohort study. Circulation.

[8] Steinberg BA (2016). Off-Label Dosing of Non-Vitamin K Antagonist Oral Anticoagulants and Adverse Outcomes: The ORBIT-AF II Registry. J. Am. Coll. Cardiology.

[9] Sørensen Rikke, Gislason Gunnar, Torp-Pedersen Christian, Olesen Jonas Bjerring, Fosbøl Emil L, Hvidtfeldt Morten W, Karasoy Deniz, Lamberts Morten, Charlot Mette, Køber Lars, Weeke Peter, Lip Gregory Y H, Hansen Morten Lock (2013). Dabigatran use in Danish atrial fibrillation patients in 2011: a nationwide study. BMJ Open.

[10] Basaran O (2016). Suboptimal use of non-vitamin K antagonist oral anticoagulants: Results from the RAMSES study. Med..

[11] Hori M (2012). Rivaroxaban vs. warfarin in Japanese patients with atrial fibrillation - the J-ROCKET AF study. Circulation J.: Off. journal Japanese Circulation Soc..

[12] Chan YH (2016). Cardiovascular, Bleeding, and Mortality Risks of Dabigatran in Asians With Nonvalvular Atrial Fibrillation. Stroke.

[13] Oldgren J (2014). Variations in cause and management of atrial fibrillation in a prospective registry of 15,400 emergency department patients in 46 countries: the RE-LY Atrial Fibrillation Registry. Circulation.

[14] Piccini JP (2014). Relationship between time in therapeutic range and comparative treatment effect of rivaroxaban and warfarin: results from the ROCKET AF trial. J. Am. Heart Assoc..

[15] Goto S (2014). Efficacy and safety of apixaban compared with warfarin for stroke prevention in patients with atrial fibrillation from East Asia: a subanalysis of the Apixaban for Reduction in Stroke and Other Thromboembolic Events in Atrial Fibrillation (ARISTOTLE) Trial. Am. heart J..

[16] Bijlsma MJ, Janssen F, Hak E (2016). Estimating time-varying drug adherence using electronic records: extending the proportion of days covered (PDC) method. Pharmacoepidemiology drug. Saf..

[17] Rosendaal FR, Cannegieter SC, van der Meer FJ, Briet E (1993). A method to determine the optimal intensity of oral anticoagulant therapy. Thromb. Haemost..

[18] Schulman S, Kearon C (2005). Definition of major bleeding in clinical investigations of antihemostatic medicinal products in non-surgical patients. J. Thromb. Haemost..

[19] Pazzagli L (2018). Methods for time-varying exposure related problems in pharmacoepidemiology: An overview. Pharmacoepidemiology drug. Saf..

[20] Firth D (1993). Bias Reduction of Maximum-Likelihood-Estimates. Biometrika.

[21] Laliberte F (2014). Real-world comparative effectiveness and safety of rivaroxaban and warfarin in nonvalvular atrial fibrillation patients. Curr. Med. Res. Opin..

[22] Yao, X. *et al*. Effectiveness and Safety of Dabigatran, Rivaroxaban, and Apixaban Versus Warfarin in Nonvalvular Atrial Fibrillation. Journal of the American Heart Association 5, 10.1161/jaha.116.003725 (2016). 10.1161/JAHA.116.003725 PMC4937291 27412905

[23] Lee SR (2018). Edoxaban in Asian Patients With Atrial Fibrillation: Effectiveness and Safety. J. Am. Coll. Cardiology.

[24] Chan YH (2016). Thromboembolic, Bleeding, and Mortality Risks of Rivaroxaban and Dabigatran in Asians With Nonvalvular Atrial Fibrillation. J. Am. Coll. Cardiology.

[25] Kaplan RM, Chambers DA, Glasgow RE (2014). Big data and large sample size: a cautionary note on the potential for bias. Clin. Transl. Sci..

[26] Bjorck F (2016). Outcomes in a Warfarin-Treated Population With Atrial Fibrillation. JAMA cardiology.

[27] Connolly SJ (2008). Benefit of oral anticoagulant over antiplatelet therapy in atrial fibrillation depends on the quality of international normalized ratio control achieved by centers and countries as measured by time in therapeutic range. Circulation.

[28] Hori M, Ezekowitz MD, Reilly PA (2014). Response to letter regarding article, “Dabigatran versus warfarin: effects on ischemic and hemorrhagic strokes and bleeding in Asians and non-Asians with atrial fibrillation”. Stroke.

[29] Wong KS (2014). Rivaroxaban for stroke prevention in East Asian patients from the ROCKET AF trial. Stroke.

[30] Yamashita T (2016). Edoxaban vs. Warfarin in East Asian Patients With Atrial Fibrillation- An ENGAGE AF-TIMI 48 Subanalysis. Circulation J.: Off. journal Japanese Circulation Soc..

[31] Yamaguchi T (2000). Optimal intensity of warfarin therapy for secondary prevention of stroke in patients with nonvalvular atrial fibrillation: a multicenter, prospective, randomized trial. Japanese Nonvalvular Atrial Fibrillation-Embolism Secondary Prevention Cooperative Study Group. Stroke.

[32] Yasaka M, Minematsu K, Yamaguchi T (2001). Optimal intensity of international normalized ratio in warfarin therapy for secondary prevention of stroke in patients with non-valvular atrial fibrillation. Intern. Med..

[33] Guidelines for Pharmacotherapy of Atrial Fibrillation JCS (2013). Circulation journal: official journal of the Japanese Circulation Society **78**, 1997–2021 (2014). 10.1253/circj.cj-66-0092 24965079

[34] Yao X (2017). Antagonist Oral Anticoagulant Dosing in Patients With Atrial Fibrillation and Renal Dysfunction. J. Am. Coll. Cardiology.

[35] Stangier J, Rathgen K, Stahle H, Gansser D, Roth W (2007). The pharmacokinetics, pharmacodynamics and tolerability of dabigatran etexilate, a new oral direct thrombin inhibitor, in healthy male subjects. Br. J. Clin. Pharmacol..

[36] Kubitza D, Becka M, Voith B, Zuehlsdorf M, Wensing G (2005). Safety, pharmacodynamics, and pharmacokinetics of single doses of BAY 59-7939, an oral, direct factor Xa inhibitor. Clin. Pharmacol. Ther..

[37] Frost C (2013). Safety, pharmacokinetics and pharmacodynamics of multiple oral doses of apixaban, a factor Xa inhibitor, in healthy subjects. Br. J. Clin. Pharmacol..

