# Supplementary material for: Effectiveness and Safety of Off-label Dosing of Non–vitamin K Antagonist Anticoagulant for Atrial Fibrillation in Asian Patients
Source: Sci Rep. 2020 Feb 4;10:1801. doi: 10.1038/s41598-020-58665-5 (PMC7000392; doi:10.1038/s41598-020-58665-5)

Effectiveness and Safety of Off-label Dosing of Non–vitamin K Antagonist Anticoagulant for Atrial Fibrillation in Asian Patients

Kwang-No Lee, MD[1]; Jong-Il Choi, MD, PhD, MHSc[1,*]; Ki Yung Boo, MD[1]; Do Young Kim, MD[1]; Yun Gi Kim, MD[1]; Suk-Kyu Oh, MD[1]; Yong-Soo Baek, MD[2]; Dae In Lee, MD[3]; Seung-Young Roh, MD[1]; Jaemin Shim, MD, PhD[1]; Jin Seok Kim, MD, PhD[1]; Young-Hoon Kim, MD, PhD[1]

[1]Division of Cardiology, Department of Internal Medicine, Korea University College of Medicine and Korea University Medical Center, Seoul, Republic of Korea; [2]Division of Cardiology, Department of Internal Medicine, Inha University Hospital, Incheon, Republic of Korea; [3]Division of Cardiology, Department of Internal Medicine, Chungbuk National University Hospital, Cheongju, Republic of Korea

*Corresponding author: +82-2-920-5445, jongilchoi@korea.ac.kr, Korea University Anam hospital, 73 Goryeodae-ro, Seongbuk-gu, Seoul 02841, Republic of Korea.

Supplementary Table S1. Dosage and administration of non–vitamin K antagonist anticoagulants and approval dates by the Korean Ministry of Food and Drug Safety to prevent stroke and systemic embolism associated with non-valvular atrial fibrillation

Drug	Approval date	Dosage and administration*
Dabigatran 110 mg	Feb. 18, 2011	110 mg twice daily<br>- 30 ≤serum creatinine clearance (sCCr) <50 mL/min<br>- body weight ≤50 kg<br>- age ≥75 years<br>- concomitant potent P-glycoprotein inhibitor therapy†
Dabigatran 150 mg	Feb. 18, 2011	150 mg twice daily, standard dose
Rivaroxaban 15 mg	Mar. 6, 2012	15 mg once daily<br>- 15 ≤ sCCr < 50 mL/min
Rivaroxaban 20 mg	Mar. 6, 2012	20 mg once daily, standard dose
Apixaban 2.5 mg	Jan. 8, 2013	2.5 mg twice daily<br>- any 2 of age ≥80 years, body weight ≤60 kg, or serum creatinine ≥1.5 mg/dL<br>- 15 ≤ sCCr < 30 mL/min

Apixaban 5 mg	Feb. 27, 2013	5 mg twice daily, standard dose
Edoxaban 30 mg	Aug. 25, 2015	30 mg once daily<br>- 15 ≤ sCCr < 50 mL/min<br>- body weight ≤60 kg<br>- concomitant potent P-glycoprotein inhibitor therapy‡
Edoxaban 60 mg	Aug. 25, 2015	60 mg once daily, standard dose

* based on randomized controlled trials except for dabigatran.

† included verapamil, cyclosporin, tacrolimus, dronedarone, and itraconazole in this study.

‡ included cyclosporin, tacrolimus, dronedarone, and itraconazole in this study.

Supplementary Table S2. Incidence rate and hazard ratio for thromboembolism and major bleeding compared with the all warfarin group

	Incidence rate (% patients/year)	vs. All warfarin			
		Unadjusted HR (95% CI)	*p*	Adjusted HR (95% CI)*	*p*
Thromboembolism	1.34				
All warfarin	1.35	reference	–	reference	–
All NOAC	1.52	1.04 (0.65–1.64)	0.883	1.31 (0.81–2.12)	0.273
Reduced dose	2.32	1.61 (0.96–2.71)	0.069	1.55 (0.89–2.68)	0.122
On-label use	1.94	1.34 (0.68–2.64)	0.403	1.12 (0.54–2.31)	0.760
Off-label use	2.73	1.84 (0.97–3.50)	0.063	2.51 (1.28–4.93)	0.008
Standard dose	0.92	0.61 (0.32–1.15)	0.125	0.98 (0.48–1.99)	0.952
On-label use	1.05	0.70 (0.37–1.32)	0.270	1.20 (0.58–2.49)	0.621
Off-label use	0.00	0.05 (0.00–30.86)	0.353	1.06 (0.23–4.89)	0.944†
Major bleeding	1.59				
All warfarin	2.14	reference	–	reference	–
All NOAC	1.20	0.49 (0.31–0.77)	0.002	0.53 (0.32–0.88)	0.015

Reduced dose	1.29	0.54 (0.30–0.97)	0.039	0.45 (0.23–0.87)	0.018
On-label use	1.23	0.52 (0.24–1.15)	0.107	0.31 (0.12–0.81)	0.016
Off-label use	1.46	0.59 (0.27–1.31)	0.197	0.73 (0.31–1.71)	0.467
Standard dose	1.13	0.46 (0.26–0.81)	0.007	0.65 (0.33–1.28)	0.216
On-label use	0.89	0.36 (0.19–0.69)	0.002	0.57 (0.26–1.24)	0.158
Off-label use	3.88	1.59 (0.63–3.98)	0.326	0.99 (0.30–3.25)	0.987

* adjusted for age, sex, hypertension, diabetes mellitus, congestive heart failure, prior thromboembolism, concomitant antiplatelet drugs, serum creatinine clearance, and left atrial anteroposterior diameter.

† Firth's bias reduction method was used because of sparse data.

Supplementary Table S3. Incidence rate and hazard ratio for thromboembolism and major bleeding compared with the well-controlled warfarin group

	Incidence rate (% patients/year)	vs. Warfarin with TTR ≥60%			
		Unadjusted HR (95% CI)	*p*	Adjusted HR (95% CI)*	*p*
Thromboembolism	1.34				
Warfarin with TTR ≥60%	0.92	reference	–	reference	–
All NOAC	1.52	1.36 (0.59–3.10)	0.471	1.72 (0.74–3.97)	0.206
Reduced dose	2.32	2.25 (0.94–5.42)	0.070	2.14 (0.85–5.42)	0.107
On-label use	1.94	1.79 (0.65–4.90)	0.258	1.99 (0.61–6.46)	0.251
Off-label use	2.73	2.50 (0.93–6.73)	0.070	3.12 (1.12–8.67)	0.029
Standard dose	0.92	0.75 (0.29–1.98)	0.565	1.22 (0.42–3.55)	0.711
On-label use	1.05	0.87 (0.33–2.28)	0.773	1.47 (0.49–4.40)	0.491
Off-label use	0.00	0.03 (0.00–232.19)	0.450	1.08 (0.06–18.65)	0.960†
Major bleeding	1.59				
Warfarin with TTR ≥60%	1.15	reference	–	reference	–

All NOAC	1.20	1.27 (0.52–3.15)	0.600	1.32 (0.50–3.51)	0.574
Reduced dose	1.29	1.47 (0.51–4.20)	0.473	1.30 (0.41–4.13)	0.653
On-label use	1.23	1.55 (0.46–5.23)	0.476	1.16 (0.26–5.13)	0.842
Off-label use	1.46	1.67 (0.49–5.67)	0.413	2.24 (0.62–8.17)	0.221
Standard dose	1.13	1.34 (0.48–3.72)	0.572	1.72 (0.54–5.47)	0.359
On-label use	0.89	0.93 (0.31–2.80)	0.902	1.43 (0.41–5.05)	0.574
Off-label use	3.88	6.16 (1.60–23.62)	0.008	4.53 (0.76–27.08)	0.098

* Adjusted factors were the same as given in Supplemental Table S2.

† Firth's bias reduction method was used because of sparse data.

Supplementary Table S4. Sensitivity analysis of incidence rate and hazard ratios for thromboembolism and major bleeding

	vs. All warfarin		vs. Warfarin with TTR ≥60%	
	Adjusted HR (95% CI)*	*p*	Adjusted HR (95% CI)*	*p*
Thromboembolism				
ON-R	0.93 (0.38–2.31)	0.877	1.94 (0.47–7.96)	0.358
OFF-R	2.37 (1.26–4.49)	0.008	3.19 (1.15–8.84)	0.026
ON-S	1.15 (0.55–2.37)	0.712	1.42 (0.48–4.27)	0.527
OFF-S	1.14 (0.24–5.34)	0.866†	1.26 (0.07–21.41)	0.874†
Major bleeding				
ON-R	0.28 (0.09–0.93)	0.037	1.00 (0.19–5.31)	0.996
OFF-R	0.55 (0.23–1.31)	0.176	1.78 (0.47–6.71)	0.395
ON-S	0.60 (0.29–1.27)	0.183	1.48 (0.44–5.01)	0.529
OFF-S	0.87 (0.21–3.69)	0.853	3.79 (0.50–28.67)	0.197

* Adjusted factors were the same as given in Supplemental Table S2.

† Firth's bias reduction method was used because of sparse data.

Supplementary Figure S1. Sensitivity analysis. Forest plot of adjusted hazard ratios and 95% confidence intervals for thromboembolism (a) and major bleeding (b) in the NOAC group compared with the warfarin group. TTR ≥60% was defined as well-controlled. The adjusted factors were the same as given in Supplemental Table S2.

(a) Thromboembolism

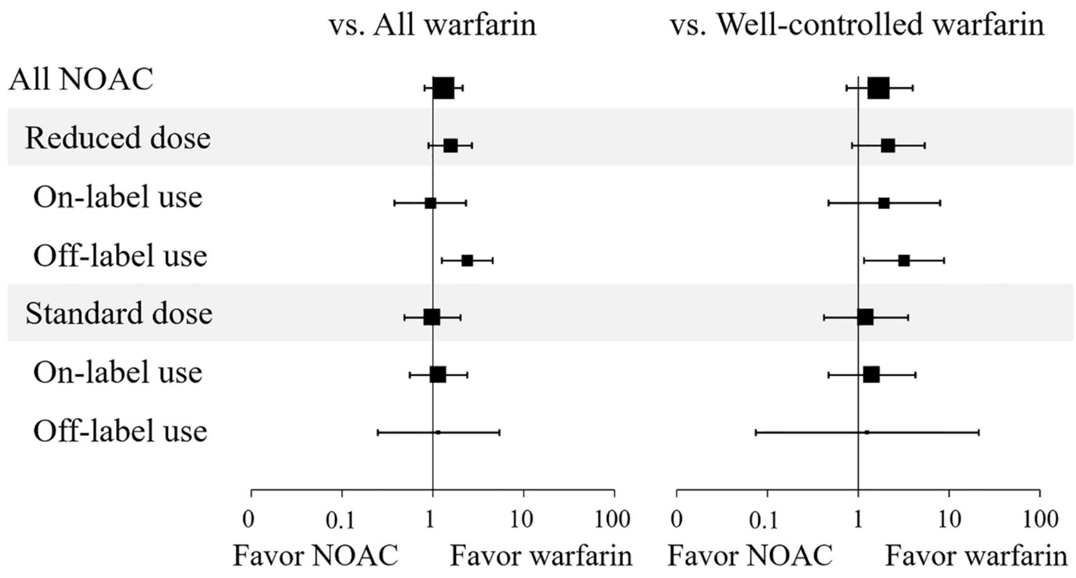

(b) Major bleeding

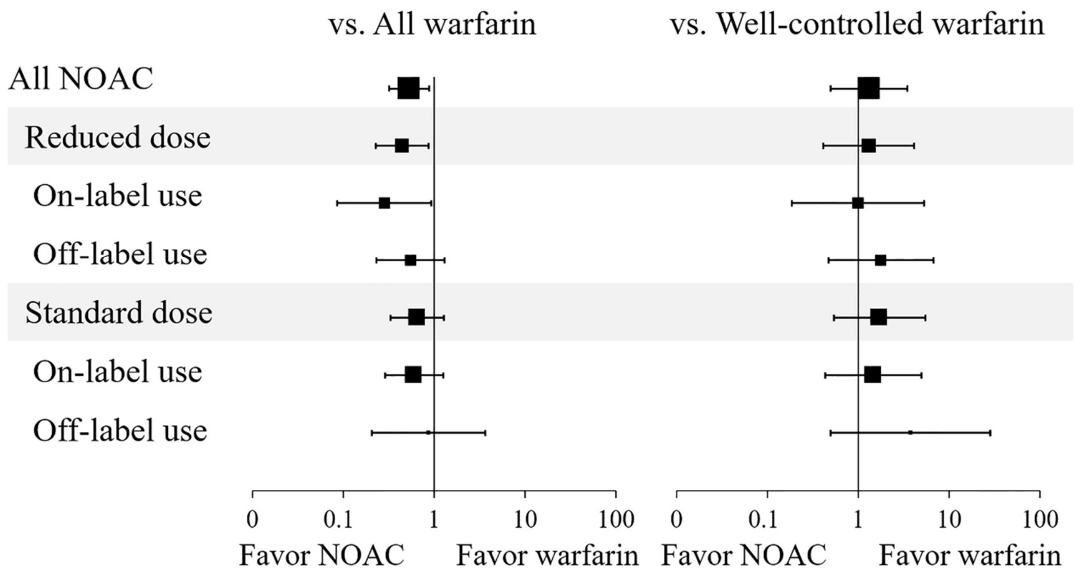

Supplement: Supplementary file 1 — Supplementary Tables and Figure. [file 41598_2020_58665_MOESM1_ESM.pdf]
